# The Role of Binge Eating in a Sequential Mediation Model of Stress, Emotional Eating, and BMI

**DOI:** 10.3390/nu17172774

**Published:** 2025-08-27

**Authors:** Kwangyeol Baek

**Affiliations:** School of Biomedical Convergence Engineering, Pusan National University, Busan 50612, Republic of Korea; kbaek@pusan.ac.kr; Tel.: +82-51-510-8548

**Keywords:** stress, emotional eating, binge eating, obesity, mediation, overeating continuum

## Abstract

**Background/Objectives:** Chronic stress contributes to obesity through maladaptive eating behaviors, including emotional eating (eating due to negative emotions) and binge eating (consuming large amounts of food with a loss of control). A theoretical model suggests that emotional eating can escalate to binge eating along a severity continuum, but this sequential pathway from stress to higher body mass index (BMI) has remained empirically untested. Therefore, this study examined a serial mediation model in which perceived stress predicts BMI sequentially through emotional eating and then binge eating. **Methods:** In this cross-sectional study, 272 Korean adults completed the Perceived Stress Scale, the Dutch Eating Behavior Questionnaire (emotional eating subscale), and the Binge Eating Scale. The serial mediation model was tested using PROCESS macro model 6, with age, gender, and education as covariates. **Results:** The serial mediation pathway (stress → emotional eating → binge eating → BMI) was statistically significant (indirect effect *B* = 0.071, 95% CI [0.041, 0.112]). A separate simple mediation path through binge eating alone was also significant (B = 0.056, 95% CI [0.018, 0.102]), whereas the path through emotional eating alone was not significant. The total indirect effect (B = 0.108, 95% CI [0.052, 0.172]) was significant, indicating that the influence of stress on BMI was fully mediated by the eating behaviors modeled. **Conclusions:** This study provides the first empirical evidence supporting a sequential pathway from stress to elevated BMI via the progression from emotional to binge eating. The findings support the overeating continuum model and highlight binge eating as a pivotal mediator. This behavioral progression suggests that emotional and binge eating are distinct stages, offering crucial opportunities for tailored prevention and intervention.

## 1. Introduction

Chronic stress contributes to obesity through multiple interconnected eating behavior changes [1]. Stress activates the hypothalamic–pituitary–adrenal (HPA) axis, leading to elevated cortisol levels that stimulate hunger and increase preference for energy-dense “comfort foods” high in fat, sugar, and calories [1,2,3]. These hormonal changes are accompanied by alterations in appetite-regulating hormones, including increased ghrelin secretion and reduced sensitivity to leptin and insulin, which collectively promote hyperphagia and disrupt normal satiety signaling [1,2,4]. Meta-analytic evidence from 54 studies (*n* = 119,820) demonstrates that stress systematically increases intake of unhealthy and energy-dense foods (Hedges’ g = 0.116) while significantly decreasing the consumption of nutritious options (Hedges’ g = −0.111) [5]. Beyond these biological processes, broader contexts shape both stress exposure and eating responses. Socioeconomic strain, weight stigma, sleep disruption, and shift work elevate allostatic load and make comfort eating more likely as a coping response [1]. Under stress, convenience-oriented food environments rich in ultra-processed foods—often observed in disadvantaged areas—bias choices toward quick and low-cost calories and away from nutritious options [6]. Importantly, consuming highly palatable foods provides temporary relief by suppressing stress responses and activating reward systems through negative feedback mechanisms that dampen HPA axis activity [1,7]. Stress also enhances brain reward pathways, particularly sensitizing the nucleus accumbens and increasing dopamine-mediated food seeking, while simultaneously impairing executive function and self-regulatory capacity [1]. This process creates a reinforcing cycle in which stress-induced eating becomes a learned coping strategy, with repeated consumption of comfort foods strengthening these maladaptive behavioral patterns [2,7]. Therefore, it is crucial to understand how stress-related changes lead to distinct maladaptive eating behaviors—especially emotional eating and binge eating—that differ in their psychological mechanisms, clinical significance, and metabolic consequences.

Among the maladaptive eating patterns, emotional eating and binge eating are recognized as critical pathways linking stress to obesity. Emotional eating is characterized by consuming food in response to emotional states rather than physiological hunger, serving as a coping mechanism to regulate negative affect such as stress, anxiety, or depression [8,9]. In contrast, binge eating involves consuming objectively large amounts of food within a discrete time period (e.g., in 2 h), accompanied by a subjective sense of loss of control over eating [10]. While emotional eating typically involves frequent episodes of modest food intake to cope with distress, binge eating reflects a more severe and dysregulated pattern of intake, often followed by intense guilt or shame [10]. Importantly, binge eating is not only more behaviorally extreme but also constitutes a core diagnostic symptom of eating disorders such as binge eating disorder (BED). These behaviors differ not only in their severity and expression but also in their neurobiological underpinnings: emotional eating is primarily associated with limbic system activation for emotion regulation, whereas binge eating is linked to prefrontal disinhibition and impaired executive control [1,10].

Emotional eating serves as a well-established mediator of the relationship between perceived stress and obesity-related outcomes [9]. For instance, in a large-scale cross-sectional study of U.S. adults, stress-related eating was found to fully mediate the link between stressful life events and key obesity markers like waist circumference and body mass index (BMI) [11]. This finding is consistent across different populations, with a study of multinational university students similarly demonstrating that emotional eating mediated the pathway from perceived stress to higher BMI [12]. The mediating role of emotional eating is also evident in high-stress populations, such as U.S. Army soldiers, where it connected perceived stress to both increased BMI and body composition failure [13]. Furthermore, this mechanism appears to influence not just weight but also dietary choices, as emotional eating partially mediated the relationship between perceived stress and poorer dietary quality in a study of Hispanic/Latino adolescents [14].

Binge eating, likewise, has been identified as another critical pathway in the stress–obesity link, with some evidence suggesting it may be a more potent mediator than emotional eating. For example, Chao and colleagues reported that although both behaviors were linked to stress, only binge eating showed a significant relationship with increased waist circumference [15]. The robustness of this pathway is strengthened by longitudinal evidence; a study of sexual-minority women demonstrated that binge eating mediated the relationship between baseline post-traumatic stress symptoms and higher BMI two years later [16]. The clinical significance of this link is further underscored in behavioral weight loss intervention studies, where eating disorder pathology mediated the effect of stress on weight loss specifically within the subgroup of patients with regular binge eating [17]. Finally, a study employing moderated mediation models clarified how this occurs, showing that stress is associated with increased likelihood of binge eating through maladaptive coping strategies [18].

Despite their distinctions, a growing body of theoretical and empirical evidence suggests that emotional eating and binge eating may lie on a single severity continuum, wherein emotional eating can progress to clinically significant binge eating [10,19]. This progression is theoretically grounded in Davis’s ‘overeating continuum model’ [19], which posits that overeating behaviors that are initially used to ‘self-medicate’ negative affect—a core functional definition of emotional eating—can escalate in severity and compulsiveness toward full-blown binge eating. This theoretical model is bolstered by converging lines of empirical evidence. For instance, a longitudinal study with adolescent girls has shown that baseline emotional eating significantly predicts the onset of binge eating symptoms over a two-year period (hazard ratio ≈ 1.8) [20]. This progression is also supported by a cross-sectional study of overweight adults, which found that emotional eating scores increase progressively from non-binge eaters to individuals with full binge eating disorder (BED) [21]. Furthermore, mechanistic insight from a study using ecological momentary assessment demonstrates that a failure to down-regulate negative affect after an emotional eating episode can create a self-reinforcing loop, precipitating full binge episodes [22]. Finally, mediation studies in adults with trauma exposure have confirmed that emotional eating is a key pathway linking post-traumatic stress to binge eating [23]. Collectively, this evidence aligns with the overeating continuum model and points to a potential sequential pathway from stress to obesity, where emotional eating develops into binge eating.

Prior research has established that both emotional eating and binge eating are independent mediators in the stress–obesity relationship. However, the sequential pathway suggested by the ‘overeating continuum model’—wherein emotional eating escalates into binge eating—has yet to be empirically tested within a single integrated framework. To address this critical gap, this study tested a serial mediation model hypothesizing that perceived stress increases BMI sequentially, first through emotional eating and then through binge eating. The present cross-sectional analysis tests a statistical model representing this hypothesized pathway, providing initial evidence. The findings are expected to help clarify the distinct roles of emotional and binge eating, potentially informing the development of more precise intervention targets. If the sequential mediation hypothesis is supported, the model could guide stage-specific strategies—stress management and emotion regulation to prevent emotional eating and evidence-based treatments for binge eating to mitigate weight-related consequences. This staging could inform person-centered screening and stepped-care approaches, improving the timing and matching of interventions for individuals at risk for stress-related eating.

## 2. Materials and Methods

### 2.1. Participants

This study was approved by the Institutional Review Boards of Pusan National University (PNU IRB 2023-18-HR and PNU IRB 2024-148-HR), and all participants provided informed consent in the present study. Participants were recruited using an IRB-approved poster that invited Korean adults to take part in an online “survey study” on eating-related concerns and food addiction. The poster specified eligibility (age: 19–49 years; exclusion: pregnancy), estimated survey duration (~60 min), and a modest honorarium provided upon completion. The poster was disseminated across university online boards, community messaging applications, and limited on-campus postings in the local area. Because this study’s primary aim was to test a theoretically specified serial mediation pathway (stress → emotional eating → binge eating → BMI) rather than estimate population prevalence, an open-call strategy was used to ensure adequate variability, including at-risk individuals, thereby increasing power to detect indirect effects; advertisements were framed as a general survey to reduce topic salience. To reduce avoidable bias and ensure data quality, we monitored the response time and included verification (bogus) items to detect inattentive or invalid responses; responses failing these checks were excluded from analyses.

Demographic information, including gender, age, years of education, height, and weight, was collected via self-report. The participants were asked to report their most recent height and weight records. Nevertheless, self-reported anthropometrics are prone to systematic under-/over-reporting, and in-person measurements would provide greater precision for BMI calculation. Of the 289 individuals initially screened, 17 participants with a BMI below 18.5 kg/m^2^ were excluded from the analysis. The final analytic sample thus comprised 272 individuals, consisting of 96 normal-weight, 39 pre-obese, and 137 obese individuals according to the diagnostic criteria in the Clinical Practice Guidelines of the Korean Society for the Study of Obesity [24]. Among these 272 participants, 115 were male (42.3%). The age distribution included 156 participants aged 29 years or younger (57.4%), 108 participants aged 30~39 years (39.7%), and 8 participants aged 40~49 years (2.9%). Participants had an average of 15.43 years of education (SD = 2.52).

A priori sample size was estimated using the Monte Carlo power analysis approach recommended by Schoemann et al. [25]. Chao et al.’s [15] correlation matrix served as the population input, and 5000 Monte Carlo replications with 95% confidence intervals were run for the serial double-mediator model. The simulation indicated that *n* ≈ 181 would yield 80% power to detect the serial indirect effect. Because the present study used different instruments for binge eating and obesity than those in the source matrix, a post hoc sensitivity analysis was conducted with identical Monte Carlo settings using the effect sizes observed in the current sample. This analysis indicated that a sample of at least 70 participants would achieve 80% power, confirming that the final sample (*n* = 272) comfortably exceeds both the a priori and post hoc requirements.

### 2.2. Clinical Questionnaires

#### 2.2.1. Perceived Stress

Perceived stress levels were measured using the 10-item Perceived Stress Scale (PSS) [26], which employs a 5-point Likert-type response format ranging from 0 to 4. Higher total scores indicate greater perceived stress. The Korean validation of the PSS yielded satisfactory internal consistency, with Cronbach’s alpha coefficients of 0.82 [27], and the present study estimated Cronbach’s alpha as 0.85.

#### 2.2.2. Emotional Eating

Emotional eating was assessed with the Dutch Eating Behavior Questionnaire (DEBQ), which comprises three subscales: emotional eating, external eating, and restrained eating [28]. Responses were recorded on a 5-point Likert-type scale from 1 (‘never’) to 5 (‘very often’), with higher scores reflecting a greater tendency toward each eating behavior. This study focused solely on the emotional eating subscale, which consists of 13 items. The Korean version of the DEBQ emotional eating subscale showed good internal consistency, with Cronbach’s alpha values of 0.92 in prior research [29] and 0.93 in this study.

#### 2.2.3. Binge Eating

Binge eating severity was evaluated using the Binge Eating Scale (BES), a widely employed 16-item self-report instrument [30]. Items are scored on either a 0–2 or 0–3 scale, depending on the item. Total scores are interpreted as follows: less than 18 indicates no binge eating symptoms; 18 to 26 indicates moderate binge eating; and 27 or above indicates severe binge eating behavior. The Korean version of the BES demonstrated good internal consistency, with Cronbach’s alpha of 0.84 in previous studies [31] and 0.90 in the present study.

### 2.3. Statistical Analysis

All statistical analyses were performed using IBM SPSS Statistics version 29.0. Descriptive statistics (means and standard deviations) and Pearson correlations were calculated for all study variables in the full sample (see Table 1). Gender was coded as a binary variable (0 = female, 1 = male) for correlation analysis.

A serial mediation model was tested using Model 6 of the PROCESS macro version 4.2 for IBM SPSS Statistics [32]. Perceived stress (PSS) was entered as the independent variable (X); emotional eating (DEBQ subscale) and binge eating (BES) were specified as mediators (M1 and M2); and BMI (kg/m^2^) was the dependent variable (Y). Gender (1 = male, 0 = female), chronological age (years), and years of education were included as covariates in the model. Indirect effects were estimated using 10,000 bias-corrected bootstrap resamples, and statistical significance was evaluated based on 95% bootstrap confidence intervals; an indirect path was considered significant when its interval did not include zero. Conventional *p*-values are reported only for the individual path coefficients in the regression models, but not for indirect effects. Unstandardized regression coefficients (B), standard errors (SE), confidence intervals, and standardized regression coefficients (β) are reported. All analyses were conducted using the full sample (*n* = 272).

## 3. Results

### 3.1. Descriptive Statistics

This study analyzed 272 participants, including 137 individuals classified as obese. Table 1 presents the descriptive statistics and correlations for all study variables in the full sample (*n* = 272). Bivariate Pearson correlation analyses showed that BMI was positively correlated with emotional eating (r = 0.182, *p* < 0.01), binge eating (r = 0.383, *p* < 0.001), and age (r = 0.214, *p* < 0.001). Perceived stress was positively correlated with both emotional eating (r = 0.426, *p* < 0.001) and binge eating (r = 0.397, *p* < 0.001). Emotional eating and binge eating were also strongly positively correlated to each other (r = 0.621, *p* < 0.001). Male gender was associated with higher perceived stress, emotional eating, and binge eating (all *p* < 0.01).

**Table 1:** Descriptive statistics of study variables (*n* = 272).

		Pearson Correlation r						
Variables	Mean (SD) or *n* (%)	1	2	3	4	5	6	7
1. Gender (female = 0, male = 1)	115 (42.3%) ^a^	-						
2. Age	28.75 (5.85)	−0.035	-					
3. Education	15.43 (2.52)	0.015	0.277 **	-				
4. BMI	25.39 (4.83)	−0.077	0.214 **	−0.025	-			
5. Perceived stress (PSS)	17.75 (6.13)	0.183 **	−0.015	−0.021	0.098	-		
6. Emotional eating (DEBQ)	33.39 (6.21)	0.254 **	0.107	0.004	0.182 **	0.426 **	-	
7. Binge eating (BES)	13.40 (8.50)	0.253 **	0.143 *	−0.058	0.383 **	0.397 **	0.621 **	-

^a^ Number and percentage of male participants are provided for gender. *: *p* < 0.05, **: *p* < 0.01 (two-tailed test).

### 3.2. Mediation Analysis

**Figure 1:**
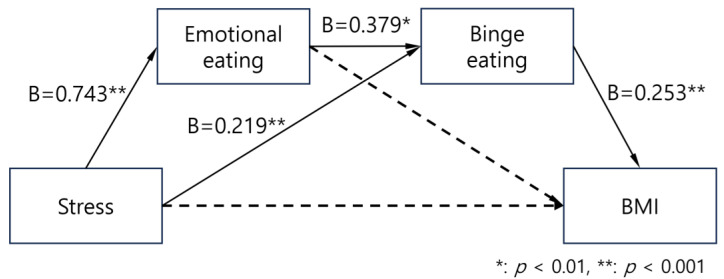
Regression coefficients in the serial mediation model (PROCESS model 6). Only significant coefficients in each path are depicted. Dashed arrows represent non-significant paths.

A serial multiple mediation analysis using PROCESS macro model 6 was performed to examine whether emotional eating and binge eating sequentially mediate the effect of perceived stress on BMI, controlling for age, years of education, and gender. Regression coefficients are presented in Table 2 and Figure 1. Perceived stress was positively associated with emotional eating (B = 0.743, SE = 0.103, *p* < 0.001), and emotional eating significantly predicted binge eating (B = 0.379, SE = 0.039, *p* < 0.001). Perceived stress also had a direct positive effect on binge eating (B = 0.219, SE = 0.071, *p* = 0.002). In the final model predicting BMI, only binge eating showed a significant positive association with BMI (B = 0.253, SE = 0.041, *p* < 0.001). The effects of perceived stress (B = −0.016, *p* = 0.738) and emotional eating (B = −0.026, *p* = 0.393) on BMI were not significant, indicating full mediation.

The total effect of perceived stress on BMI was marginally significant (B = 0.091, SE = 0.047, *p* = 0.055). However, the direct effect became non-significant after the inclusion of the mediators (B = −0.016, SE = 0.049, *p* = 0.738), indicating the presence of indirect effects.

Bootstrapping with 10,000 samples revealed a statistically significant total indirect effect of perceived stress on BMI (B = 0.108, Boot SE = 0.031, 95% CI [0.052, 0.172]; β = 0.137), as shown in Table 3. Among the three indirect pathways, the serial mediation path through emotional eating and binge eating was significant (B = 0.071, Boot SE = 0.018, 95% CI [0.041, 0.112]; β = 0.091), and the simple mediation path through binge eating alone (stress → binge eating → BMI) was also significant (B = 0.056, Boot SE = 0.022, 95% CI [0.018, 0.102]; β = 0.071). The mediation path through emotional eating alone (stress → emotional eating → BMI) was not significant (B = −0.019, Boot SE = 0.023, 95% CI [−0.068, 0.025]; β = −0.024). These results indicate that perceived stress indirectly contributes to increased BMI via its effects on binge eating, both directly and through emotional eating.

## 4. Discussion

### 4.1. Main Findings and Theoretical Implications

The present study offers the first empirical test of a serial mediation model linking perceived stress to BMI through a sequential pathway involving emotional eating and binge eating. The findings highlight two key patterns: (1) a significant serial mediation pathway (perceived stress → emotional eating → binge eating → BMI) that supports the overeating continuum hypothesis and (2) an additional direct pathway from stress through binge eating alone to BMI. Notably, emotional eating did not independently mediate BMI when controlling for binge eating, suggesting that its influence on weight may primarily operate through progression to more severe dysregulated eating behaviors. This pattern matches a theoretical continuum that places emotional eating before binge eating [19] and a review noting convergent trends for that progression, although direct evidence remains limited [10]. To be clear, the observed serial mediation should be interpreted as a statistical decomposition conditional on an assumed temporal order, not as evidence of causal progression from emotional to binge eating, particularly given the cross-sectional design of this study.

These findings provide important empirical support for Davis’s overeating continuum model [19], which conceptualizes eating behaviors along a spectrum of severity—from occasional overeating to compulsive binge eating. The significant serial mediation observed here indicates that emotional eating may serve as a developmental precursor to binge eating. This progression aligns with affect regulation models, where repeated use of food to manage negative emotions can lead to more severe loss-of-control episodes over time. The present results extend previous longitudinal findings by Stice et al. [20], who demonstrated that emotional eating prospectively predicted binge eating onset by providing direct statistical evidence within a mediation framework. Furthermore, our findings complement cross-sectional observations by Ricca et al. [21], who demonstrated stepped increases in emotional eating scores from non-binge eaters to subthreshold and full BED groups, illustrating how this progression may contribute to weight-related outcomes.

The cross-sectional questionnaire-based design in this study limits causal and temporal inferences about stress, emotional eating, and binge eating. Next steps should leverage ambulatory approaches—ecological momentary assessment, passive sensing, and food-logging apps—to capture stress, affect, cues, and intake in real time and to model within-person dynamics (e.g., lagged effects and escalation from emotional to binge eating). For example, daily longitudinal data showing that negative mood coincides with emotional eating urges—and that such urges predict worse mood the following day—strengthen the hypothesis that emotional eating is a proximal pathway toward binge eating risk [22]. Integrating wearables (heart rate variability, sleep, and activity) with geolocation and food environment exposure could test context-dependent risk periods and mechanisms hypothesized in the overeating continuum model. These designs would also reduce recall bias and improve the ecological validity of intervention targets [33,34].

The identification of a significant direct mediation pathway from stress through binge eating to BMI, independent of the serial pathway, carries important theoretical and clinical implications. This finding indicates that binge eating may develop in response to stress via multiple mechanisms, not solely through the escalation of emotional eating. Consistent with this view, prior work has linked stress-related psychopathology—such as PTSD symptoms—to subsequent increases in binge eating and weight outcomes, indicating a more direct stress-to-binge pathway [16]. Binge eating may develop more readily in response to stress among individuals characterized by person-level vulnerabilities, including genetic predispositions, attention-deficit/hyperactivity disorder, and elevated impulsivity or reward sensitivity; these risk factors have been linked to binge eating spectrum conditions across diverse samples [35,36,37]. This dual-pathway model aligns with Chao et al. [15], who reported that although both emotional eating and binge eating were linked to stress, only binge eating demonstrated a significant association with adverse metabolic outcomes. The simultaneous existence of both serial and direct pathways underscores binge eating as a critical intervention target, as it functions as the final common pathway for stress-related weight gain, regardless of whether individuals progress through emotional eating or develop binge eating independently.

### 4.2. Clinical and Practical Implications

The serial mediation results offer key guidance for prevention and intervention. First, recognizing emotional eating as a precursor to binge eating reveals a critical window for early prevention. The serial mediation findings have significant implications for prevention and intervention strategies. The identification of emotional eating as a precursor to binge eating [20,21,23] suggests a critical window for early intervention (or prevention). Early interventions targeting emotion regulation skills, stress management, and adaptive coping strategies may prevent the escalation from emotional eating to binge eating [38,39]. Second, these results underscore the need to tailor weight management interventions to behavior severity. Individuals exhibiting binge eating may benefit from intensive and specialized treatments, whereas those with predominant emotional eating could respond well to lower-intensity programs. For those with established binge eating, interventions must target both emotional triggers and loss-of-control episodes. Integrating Dialectical Behavior Therapy, mindfulness techniques, and Cognitive Behavioral Therapies can effectively address both emotion regulation deficits and binge eating behaviors [40,41,42,43].

### 4.3. Limitations

Several limitations should be acknowledged. First, the cross-sectional design prevents establishing temporal precedence among stress, emotional eating, binge eating, and BMI. Consequently, the mediation model describes conditional associations under an assumed ordering; it does not test causal effects or developmental progression. Second, the relatively small sample (*n* = 272) drawn from a single cultural context—predominantly young Korean adults—limits external validity to older, non-Korean, or clinical populations. Third, the open-call recruitment likely increased topic salience and self-selection, yielding a younger sample with higher obesity prevalence and some gender imbalance. Despite adjustment for age, gender, and education, these selection features may constrain external validity; accordingly, inferences are most applicable to online/community-recruited samples with similar age, gender, and weight distributions. Fourth, reliance on self-report questionnaires introduces potential response bias and measurement error; objective assessments (e.g., ecological momentary assessment and clinician-administered interviews) should be incorporated in future studies. Fifth, only one serial mediation model based on the overeating continuum framework was tested; alternative or parallel pathways (e.g., involving sleep disturbance, physical activity, or dietary quality) remain unexamined and warrant comparison using model comparison approaches. Sixth, unmeasured confounding is possible because we did not assess key factors that influence both BMI and eating behaviors (e.g., physical activity, socioeconomic status and food access, and depressive/anxiety symptoms); thus, estimates and mediation paths should be interpreted cautiously.

### 4.4. Future Directions

Future research should pursue the following directions:

- Longitudinal multi-wave designs are essential to establish temporal precedence and examine, via time-lagged mediation, how stress initiates emotional eating that progresses to binge eating, with these sequential changes prospectively predicting weight gain trajectories.
- Expand recruitment to larger and more diverse samples using quota- or probability-panel sampling. Conduct multigroup and weighted analyses to assess generalizability across age, gender, weight status, and ethnicity.
- Incorporate objective measures for both core constructs and confounders. For core constructs, include biological markers (e.g., cortisol) and standardized laboratory eating assessments; for confounders, consider physical activity and sleep monitoring, socioeconomic indices, food access measures, and clinician-rated depression/anxiety. These additions will reduce self-report bias and residual confounding and provide stronger mechanistic inference.
- Testing of competing mediation models and potential moderators (e.g., personality traits, genetic polymorphisms, and environmental factors) should delineate alternative pathways and identify individual vulnerability factors.
- Prospective or retrospective studies in clinical eating disorder cohorts can pinpoint transition points from emotional eating to BED, elucidate risk and protective factors, and inform targeted prevention and intervention strategies.

## 5. Conclusions

This study provides the first empirical support for a serial mediation pathway from perceived stress to higher BMI via emotional eating and subsequently binge eating while also identifying a direct stress → binge eating → BMI route; together, these findings support continuum-based models positioning emotional eating as an earlier stage that can progress to more severe binge episodes and underscore binge eating as a pivotal target for obesity prevention and treatment. Practically, emotional eating may serve as an early warning indicator warranting timely emotion regulation interventions, whereas individuals already experiencing binge eating may require more intensive and specialized care. Future work should use longitudinal multi-wave designs with larger and culturally diverse samples and incorporate objective assessments to clarify temporal ordering and strengthen the generalizability of the stress–eating–obesity pathway.

## Figures and Tables

**Table 2:** Regression coefficients for the serial mediation model (PROCESS model 6).

Outcome	Predictor	B	SE	β	*t*	*p*-Value	95% CI of B
Emotional eating	Perceived stress	0.743	0.103	0.393	7.192	<0.001	[0.540, 0.946]
	Age	0.251	0.111	0.127	2.270	0.024	[0.033, 0.470]
	Education	−0.121	0.257	−0.026	−0.470	0.639	[−0.626, 0.385]
	Gender: Male	4.378	1.281	0.187	3.418	0.001	[1.856, 6.899]
Binge eating	Perceived stress	0.219	0.071	0.158	3.079	0.002	[0.079, 0.360]
	Emotional eating	0.379	0.039	0.517	9.811	<0.001	[0.303, 0.455]
	Age	0.172	0.071	0.119	2.439	0.015	[0.033, 0.311]
	Education	−0.308	0.162	−0.091	−1.898	0.059	[−0.628, 0.011]
	Gender: Male	1.688	0.826	0.098	2.042	0.042	[0.061, 3.315]
Body mass index	Perceived stress	−0.016	0.049	−0.021	−0.335	0.738	[−0.112, 0.080]
	Emotional eating	−0.026	0.030	−0.062	−0.855	0.393	[−0.086, 0.034]
	Binge eating	0.253	0.041	0.445	6.136	0.000	[0.172, 0.334]
	Age	0.135	0.048	0.163	2.800	0.005	[0.040, 0.229]
	Education	−0.080	0.110	−0.042	−0.724	0.470	[−0.296, 0.137]
	Gender: Male	−1.597	0.560	−0.164	−2.850	0.005	[−2.700, −0.494]

**Table 3:** Indirect effects of perceived stress on BMI via emotional and binge eating (10,000 bootstrap samples).

Indirect Path	Effect (B)	Boot SE	Boot LL	Boot UL
Perceived stress → Emotional eating → BMI	−0.019	0.023	−0.068	0.025
Perceived stress → Binge eating → BMI	0.056	0.022	0.018	0.102
Perceived stress → Emotional eating → Binge eating → BMI	0.071	0.018	0.041	0.112
**Total Indirect Effect**	0.108	0.031	0.052	0.172

## Data Availability

The raw data supporting the conclusions of this article will be made available by the author upon request.

## Abbreviations

The following abbreviations are used in this manuscript:

BED	Binge eating disorder
BES	Binge Eating Scale
BMI	Body mass index
DEBQ	Dutch Eating Behavior Questionnaire
HPA	Hypothalamic–pituitary–adrenal
PSS	Perceived Stress Scale
SD	Standard deviation
